# An asymmetric variation of hot and cold SST extremes in the China Seas during the recent warming hiatus period

**DOI:** 10.1038/s41598-020-79854-2

**Published:** 2021-01-21

**Authors:** Yan Li, Qingyuan Wang, Qingquan Li, Yiwei Liu, Yan Wang

**Affiliations:** 1 College of Life Sciences and Oceanography, Shenzhen University, Shenzhen, China; 2 Tianjin Meteorological Observatory, Tianjin, China; 3 Laboratory for Climate Studies, National Climate Center, China Meteorological Administration, Beijing, China; 4 Tianjin Modification Weather Office, Tianjin, China

**Keywords:** Climate change, Ocean sciences, Physical oceanography

## Abstract

Extreme sea surface temperatures (SSTs) attract much attention in recent years. However, the detailed spatial and temporal pattern of the extreme SSTs in China Seas has not been well understood. Using the daily SST data set of OISST v2 from January 1, 1982 to December 31, 2013, and based on four extreme SST indices, the frequency and intensity of SST extremes in the China Seas were examined. The analysis showed that the annual mean SST exhibited cooling trend, on pace with a trend of − 0.34 °C/decade during 1998–2013, confirming the previous studies that China Seas also experienced the recent global warming hiatus. But during this recent global warming hiatus, there was a notable asymmetric pattern of greater cooling trends in cold SSTs as compared to the hot SSTs in this region. During 1998–2013, the cold days (CDs) frequency increased significantly by 13 days per decade and cold SST extremes which were below the 10th percentile of each year (SST_10p_) notably decreased by 0.4 °C per decade. Hot days (HD) and hot SST extremes which were above the 90th percentile of each year (SST_90p_) slowed down, but without any distinct tendency. Meanwhile, the rates of SST_10p_ and CDs were highly heterogeneous in space. Cold extremes in the near-shore areas are much more sensitive to the global warming hiatus than these in the eastern of the Kuroshio Current. Importantly, hot extremes do not reveal any distinct cooling tendency during 1998–2013, there were more frequent hot days and more intense hot SSTs in this region comparing with 1982–1997. These hot extremes could push some marine organisms, fisheries and ecosystems beyond the limits of their resilience, with cascading impacts on economies and societies.

## Introduction

The globally averaged combined land and ocean surface temperature has experienced a significant acceleration in warming from the early-1980s to the later-1990s^1–3^, but has nearly stalled or paused during approximately 1998–2013, a phenomenon always call ‘global warming hiatus’^4^. Considering the continuous increase in anthropogenic greenhouse gas concentration, it is hard to explain the slowdown of globally temperature in this period. This phenomenon has attracted more attention from the climate research communities^5–8^. Researchers have noted that the “global warming hiatus” is not a worldwide phenomenon but mainly due to exceptionally cold events over Northern Hemisphere (NH) continents during boreal winter^9,10^. Researchers from China confirmed that the mainland China has experienced a remarkable cooling since 1998 under the global warming hiatus^10–12^. The China Seas is an important marginal sea located between the mainland China and the northwest Pacific Ocean with rich marine ecosystems and resources. Recent study found that the China Seas also experienced a remarkable declining trend during the global warming hiatus^13^.

Extreme events can be defined normally based on the frequency distribution of observations recorded at a location, and extreme-values are lying in the upper or lower location of the probability distribution function and would thus be rare events^14^. Over the past decades, extreme weather and climate events have caused great concern due to their huge impacts on human society and ecosystems. As with extreme events on land, oceanic extreme events including intense upwelling, deoxygenation events, extreme sea level and marine heatwaves can affect the near-surface oceans with a range of impacts for marine life and dependent communities, causing shifts in species ranges, local extinctions, and economic loss on aquaculture and seafood industries through declines in important fishery species^15–18^. Shen et al.^19^ found that the recent warming hiatus in China was reflected by cooling of cold extremes, but hot extremes do not reveal any pause in its warming pace. Few studies have paid attention to the changes of water temperature extremes in the China Seas during the recent global warming hiatus. Annual mean SST has experienced warming hiatus in the China Seas since 1998^13,20^, whether and how these extremes changed during the recent warming hiatus remains unclear. The purpose of this study is to explore the patterns of the changes in extremes in the coastal China seas and adjacent waters (hereafter referred to as the China Seas), particularly for the recent hiatus period of 1998–2013. Results are of great significance to understand the past and present climate change and predict future change of SST extremes in this region.

## Dataset

The 0.25° × 0.25° Daily SST data are from the NOAA Optimum Interpolation SST v2 (OISST v2), covering from 1th January 1982 to 31th December 2013^21^ (available online: www.esrl.noaa.gov/psd/data/gridded/data.noaa.oisst.v2.highres.html). OISST v2 uses Advanced Very High Resolution Radiometer (AVHRR) infrared satellite SST data from the Pathfinder satellite and also incorporates in situ observations, buoy data, ship data, and sea ice data^22^. As the first availability of global satellite SST, this data set has been widely used in many researches for the detection of SST extremes^23–25^. The period of 1982–2013 was separated into two periods (1982–1997, hereafter called global warming period; 1998–2013, called global warming hiatus) for analysis and comparison.

**Figure 1:**
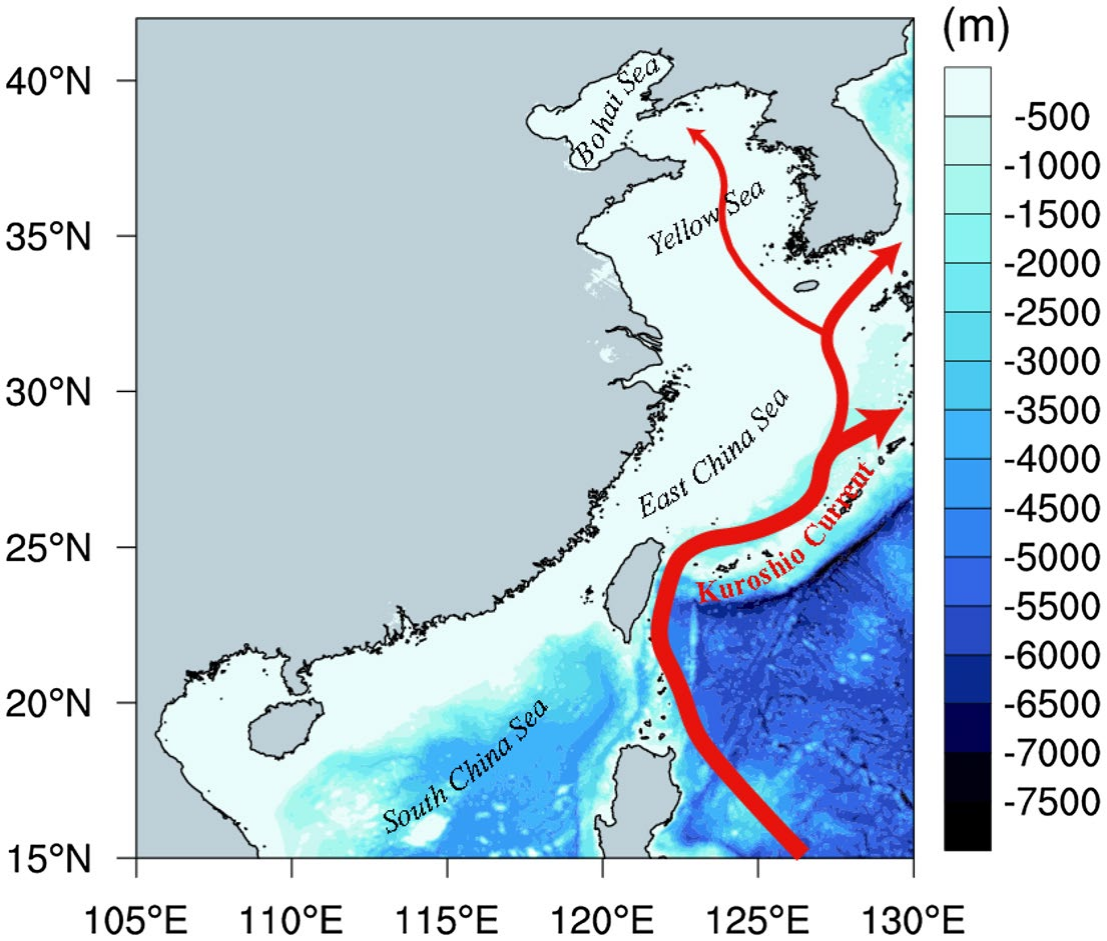
Map of the study area in the China Seas and its adjacent seas with bathymetry (Shaded areas) and marked locations of the Bohai Sea, the Yellow Sea, the East China Sea and the South China Sea. The schematic pathway of the Kuroshio Current is shown in red line. The map was created by the authors using NCAR Command Language software Version 6.6.2 (see http://dx.doi.org/10.5065/D6WD3XH5).

The China Seas is located at the south-eastern part of Asian continental shelf and the western edge of the North Pacific Ocean, including the Bohai Sea, the Yellow Sea, the East China Sea (ECS) and the South China Sea (SCS). The Kuroshio Current is the most important western boundary current in this area (Fig. 1). The coastal China Seas has a variety of special coastal ecosystems including coral reef, mangroves, tidal marshes, seagrass meadows, and form many marine biological communities and commercial fisheries areas. It is of great significance for the sustainable development of society and economy in coastal China. This vital region is greatly influenced by global climate change through the East Asian monsoon, run-off from major rivers, the Kuroshio Current and anthropogenic activities^26–28^. Many coastal ecosystem disasters and serious environmental stress, caused by SST extreme events, were reported even during the recent global warming hiatus^29–31^. The task of understanding the coastal SST variations within the context of global climate change is an urgent matter. Thus, the study area in our work is focused on the coastal China Seas and its adjacent seas with the approximate range of (15°–45°N, 105°–130°E) (Fig. 1).

## Results

**Figure 2:**
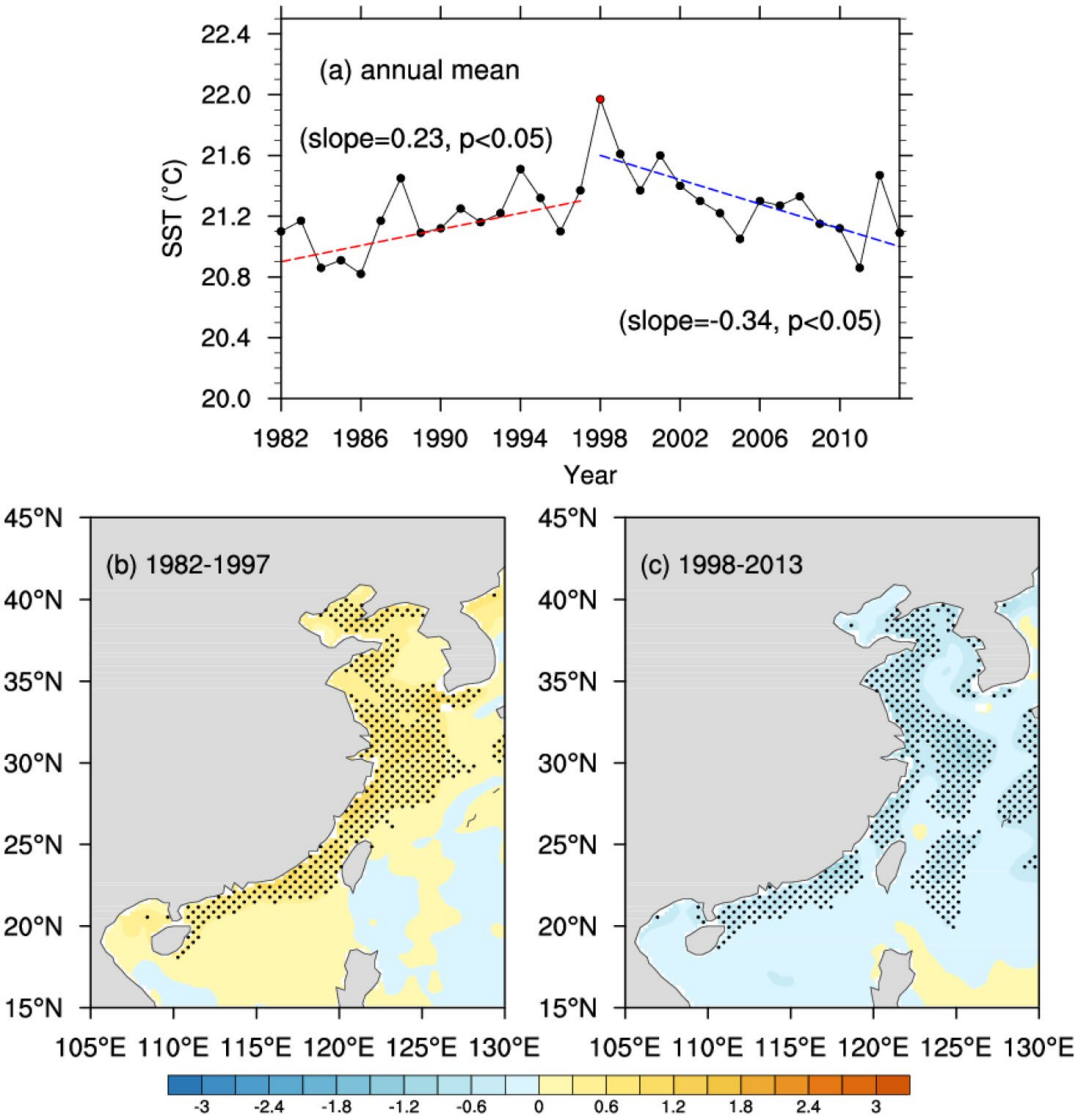
The temporal variations of annual mean SST (**a**). The red dot represents the year of 1998, the red dashed line represents the linear trend in the warming period and the blue dashed line represents the linear trend in the hiatus period. Spatial distribution of trends of annual mean SST during 1982–1997 (**b**), 1998–2013 (**c**), where the black dots represent the trends are significant at 95% confidence level (*p* < 0.05) using the Mann–Kendall test. (Units: °C per decade). Maps on panels (**b**,**c**) were created by the authors using NCAR Command Language software Version 6.6.2 (see http://dx.doi.org/10.5065/D6WD3XH5).

The time series of regional average temperatures showed fluctuant variations from 1982 to 2013. Using the cumulative sum test and bootstrap analysis, we detected a significant (exceeding 95% confidence level) temporal breakpoint at 1998 in this time series. This breakpoint divides the whole period into two periods, with a significant warming trend of 1982–1997 (0.23 °C/decade) and notable cooling trend of 1998–2013 (− 0.34 °C/decade) (Fig. 2a). Then, we showed the rates spatial distribution of annual mean SSTs during 1982–1997 and 1998–2013 in Fig. 2b,c, respectively. In the warming period (1982–1997), shallow nearshore area of the China Seas has experienced significant rapid warming (Fig. 2b). In the hiatus period, decreasing trends of annual mean SST have been detected in most part of the coastal China Seas (Fig. 2c). The coastal China Seas with shallow water depth experienced a conspicuous shift from a notable warming tendency to cooling tendency. However, these shifts were not spatially uniform and there were almost no notable shifts in the east of the Kuroshio Current with deep water depth. In the context of the recent global warming hiatus, the SST at the nearshore areas were much more sensitivity than the SST at the east of the Kuroshio Current.

**Figure 3:**
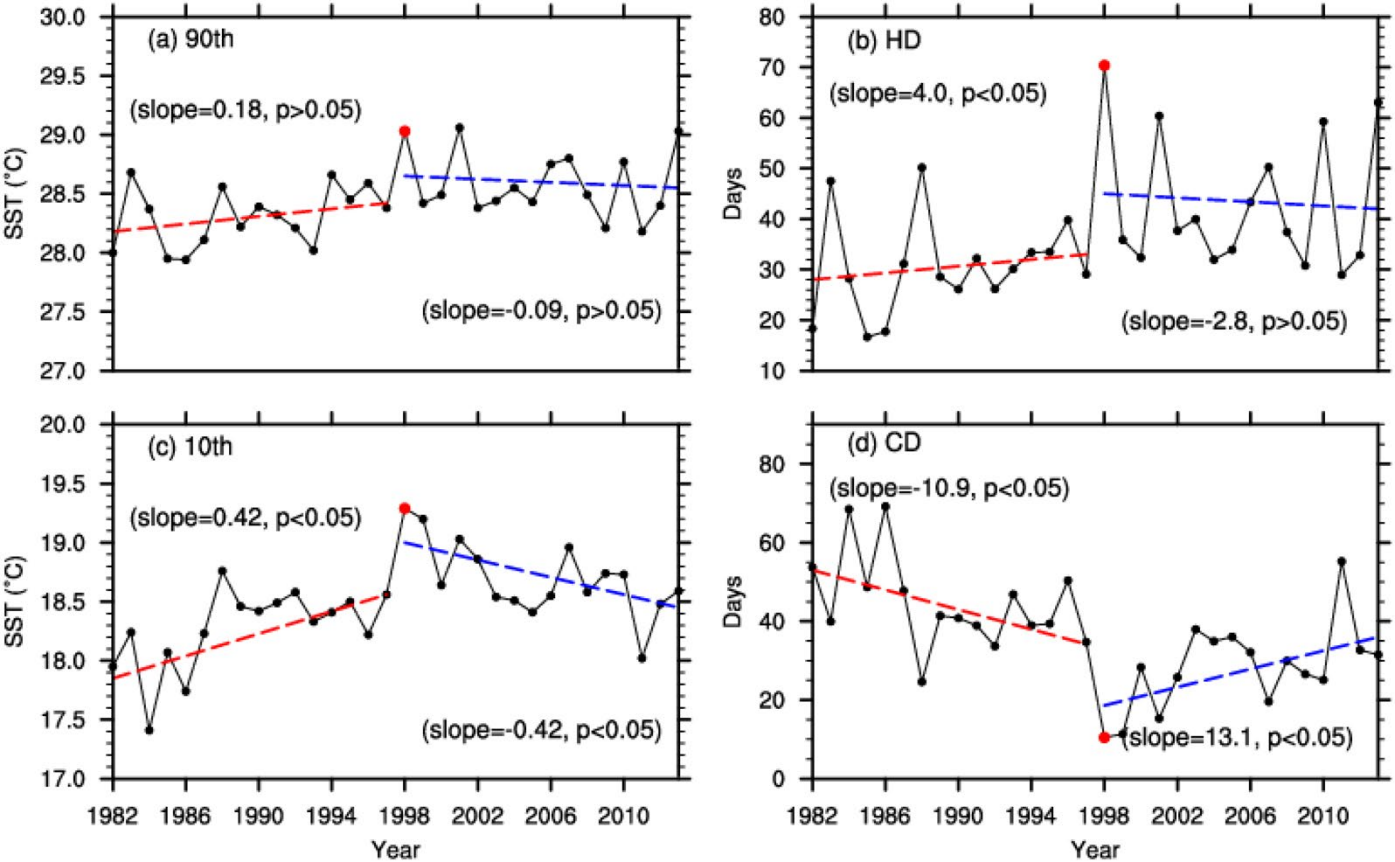
Temporal variations of regionally averaged SST_90p_ (**a**), SST_10p_ (**b**), CDs (**c**), HDs (**d**), from 1982 to 2013. The red dots represent the year of 1998, the red dashed lines represent the linear trend in the warming period and the blue dashed lines represent the linear trend in the hiatus period (Units: °C per decade).

In this part, we estimated trends of the four SST indices shown in Table 1. The time series of regional averaged SST indices (SST_90p_, SST_10p_, HDs and CDs) in each year showed fluctuant variations from 1982 to 2013 (Fig. 3). Because of the shortness of the warming period and hiatus period, significance testing of the trends has limited relevance. For hot SSTs (both SST_90p_ and HDs), the magnitudes of increasing tends were 0.18 °C per decade (non-significant at 95% confidence level, *p* > 0.05) and 4 days per decade during 1982–1997, respectively. For SST_10p_ exhibited significant increasing tendency by 0.42 °C per decade and CDs exhibited decreasing tendency by 11.0 days per decade during 1982–1997, consistent with global warming (both statistically significant at 95% confidence level, *p* < 0.05). From 1998 to 2013, hot extremes showed insignificant decreasing, with a high level of uncertainty (non-significant at 95% confidence level, *p* > 0.05). In contrast, cold extremes exhibited a significant decline (both significant at 95% confidence level, *p* < 0.05). Results confirmed that the asymmetric pattern of greater cooling trend in cold SSTs in the coastal China Seas during the recent warming hiatus period as compared to the hot SSTs (− 0.42 versus − 0.09 °C per decade; 13.1 versus − 2.8 days per decade). Such asymmetric changes in hot and cold temperatures have been confirmed in many other studies^32–34^, but with few studies focus on water temperatures.

**Table 1:** Abbreviation and definition of the indices of extremes in this study.

Variable	Abbreviation (unit)	Definition of extreme indices
Sea surface temperature (SST)	SST_90P_ (℃)	Daily SST above the 90th percentile of each year
	SST_10P_ (℃)	Daily SST below the 10th percentile of each year
	HD (days)	Numbers of days in each year when daily SST > 90th percentile of 1983–2012 baseline period
	CD (days)	Numbers of days in each year when daily SST < 10th percentile of 1983–2012 baseline period

**Figure 4:**
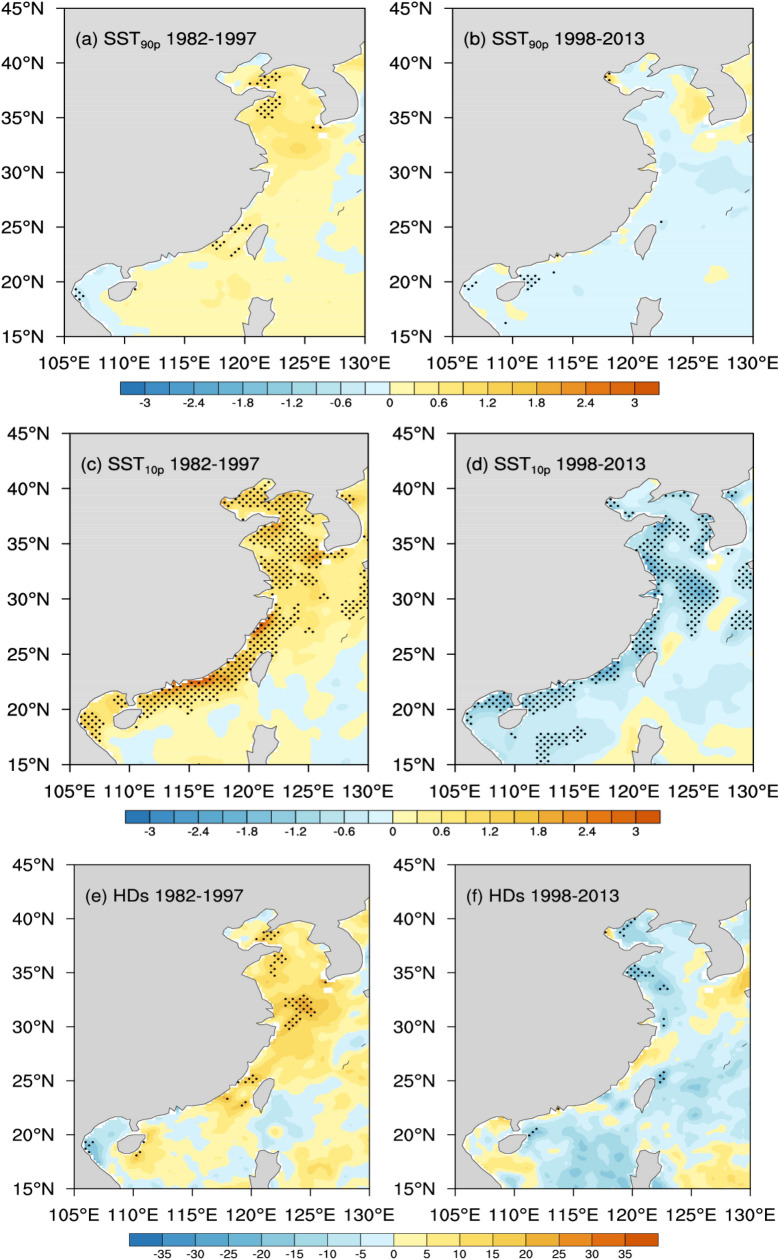

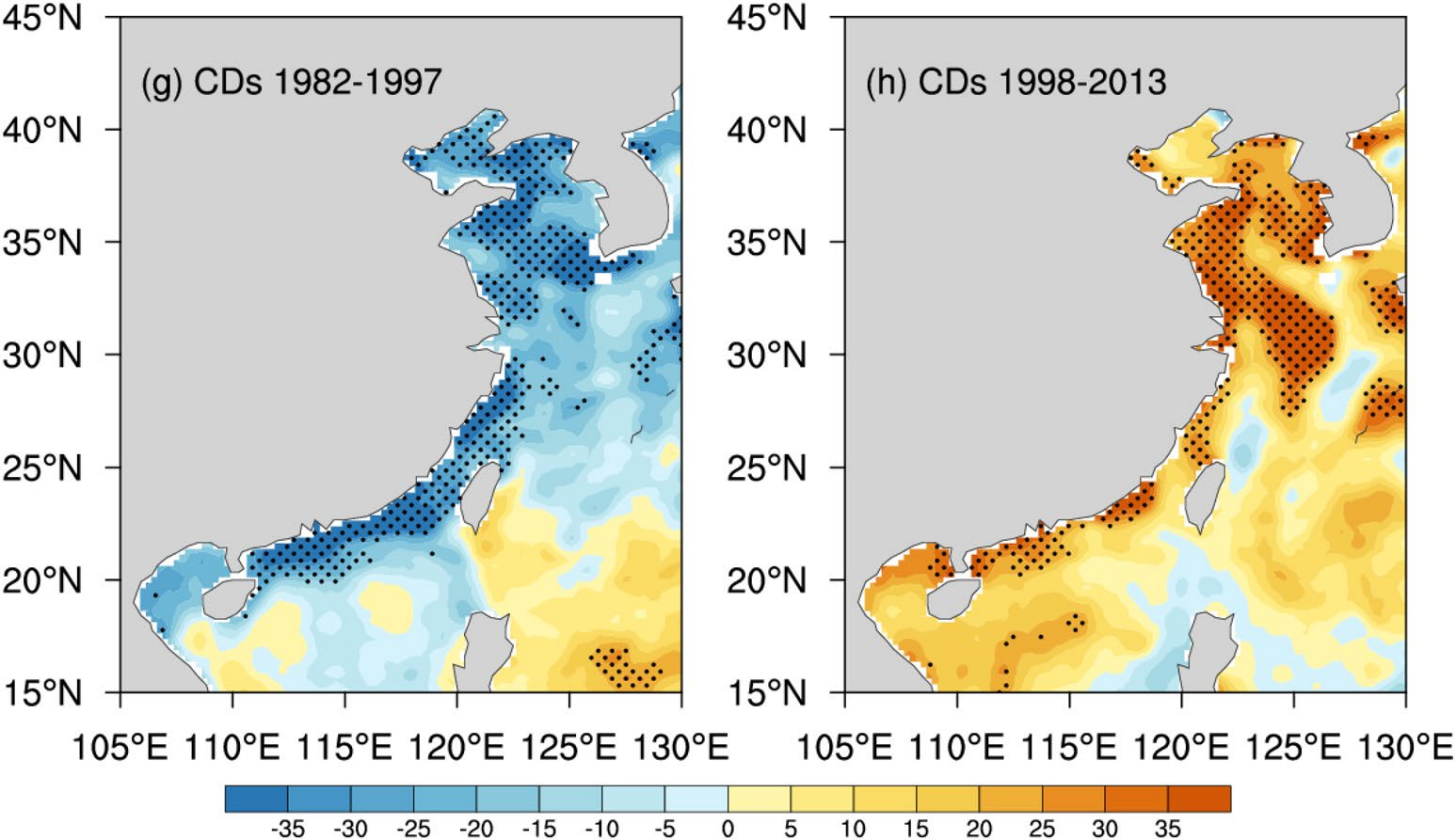
Annual trends of SST_90p_ (°C per decade), SST_10p_ (°C per decade), HDs (days per decade) and CDs (days per decade), in the coastal China Seas for the period 1982 to 1997 (**a**,**c**,**e**,**g**) and 1998 to 2013 (**b**,**d**,**f**,**h**), where the stippling indicates a 95% confidence level (*p* < 0.05) using the Mann–Kendall test (Units: °C per decade). Maps on panels (**a**–**h**) were created by the authors using NCAR Command Language software Version 6.6.2 (see http://dx.doi.org/10.5065/D6WD3XH5).

Figure 4 shows the spatial distributions of the rates of hot and cold extremes in the coastal China Seas. During 1982–1997, both SST_90p_ and HDs have increased significantly only in parts of the Bohai and Yellow Seas (Fig. 4a,e). During 1998–2013, hot extremes decreased slightly (trends very close to zero or zero) (Fig. 4b,f). In contrary, SST_10p_ increased significantly in the most part of the west of the Kuroshio Current, exceeding 0.8 °C per decade, especially in the near-shore areas with shallow depth, exceeding 1.2 °C per decade (Fig. 4c). The CDs declined significantly by about − 3 ~ 5 days per decade, with the larger rates of decline also in the near-shore areas (Fig. 4g).

During 1998–2013, SST_10p_ decreased significantly in the most part of the western Kuroshio Current, exceeding 0.7 °C per decade, especially in the near-shore areas, exceeding 1.2 °C per decade (Fig. 4d). The CDs increased significantly by about 3–5 days per decade, especially in the near-shore areas (Fig. 4h). For cold extremes, both SST_10p_ and CDs revealed warming trends during 1982–1997 but showed strikingly opposite cooling trends after 1998 in the majority of the west of the Kuroshio Current, particularly in the shallow regions (Fig. 4c–h). For hot extremes, there were not remarkable tendency shifts in the both periods (Fig. 4a–f).

Results suggest that during the warming hiatus period, there was substantial decreases tendency in the cold extremes (when the temperature decreases, the frequency of CDs increases, and vice versa) and hot extremes almost have no notable tendency. This conclusion is supported by time series of annual extreme SSTs averaged in the study regions shown in Fig. 3. Li et al.^10^ and Shen et al.^19^ reported that the recent warming hiatus with respect to mean and extreme temperatures were found in mainland China which may be related to the change of atmospheric circulations in winter. Based on our results, we confirmed that the seasonal temperature tendency differences after 1998 was also in ocean temperatures in the China Seas. The trends of cold extremes in the both periods display substantial spatial heterogeneity. The notable tendency shifts appeared in the west of the Kuroshio Current, especially in the near-shore areas with shallow water depth.

**Figure 5:**
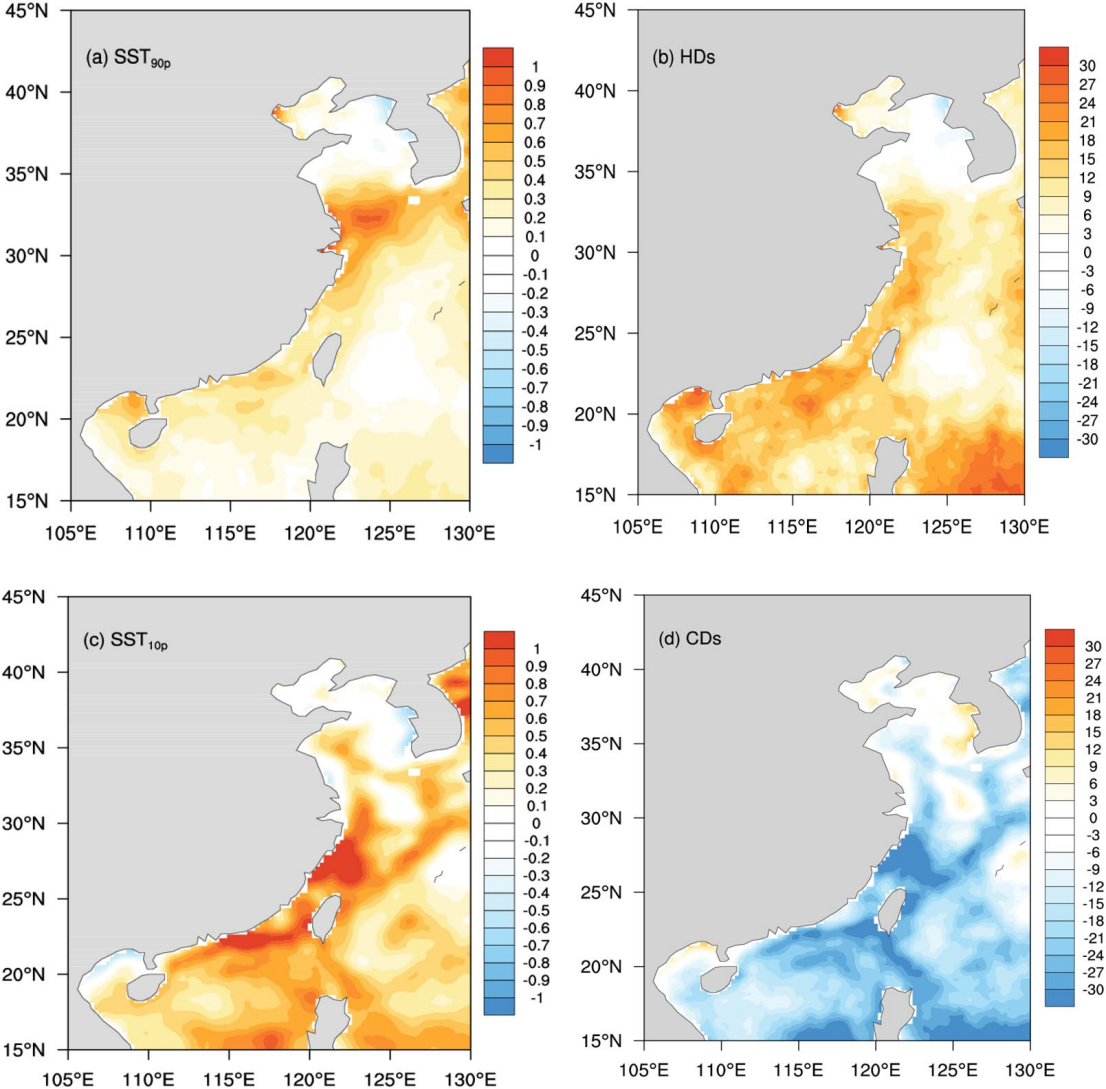
Spatial differences of SST_90p_ (°C) (**a**), SST_10p_ (°C) (**b**), HDs (days) (**c**) and CDs (days) (**d**) for the periods of 1998–2013 minus 1982–1997. Maps on panels (**a**–**d**) were created by the authors using NCAR Command Language software Version 6.6.2 (see http://dx.doi.org/10.5065/D6WD3XH5).

Interestingly, as shown in Fig. 3, although regional averaged SST_90p_ experienced non-significant cooling tendency and SST_10p_ experienced a significant cooling tendency after 1998, all of these extreme values during 1998–2013 were still higher than these during 1982–1997. The distributions of the differences of SST_90p_ and SST_10p_ between the 1998–2013 and 1982–1997 periods are displayed in Fig. 5a,c, respectively. There are regionally varying positive temperature differences in the most part of the coastal China Seas in Fig. 5. For SST_90p_, the larger differences are mostly in the Yangtze River Estuary (30°–35°N, 120°–125°N), with the center value of ~ 0.8 °C (Fig. 5a). For SST_10p_, the larger differences are located in the coastal SCS and the west of the Kuroshio Current in the ECS, with the center value exceeding 1.0 °C. Thus, the temperature changes in the two periods were spatially non-uniformed. And showed an asymmetric character through the year, with the larger differences occurred in wintertime. For HDs and CDs, the differences between the two periods are opposite (Fig. 5b,d). Compared with 1982–1997, the frequency of HDs increased in most part of the study region (except the Bohai Sea and the Yellow Sea) during 1998–2013, while the frequency of CDs significantly reduced. Importantly, Although SST_90p_ and HDs experienced insignificant decreasing tendency during 1998–2013, there were still more intense and frequent hot extremes in the coastal China Seas Importantly, hot extremes do not reveal any distinct cooling tendency during 1998–2013, there were more frequent hot days and more intense hot SSTs in this region comparing with 1982–1997 (Fig. 5a,b).

## Conclusion and discussion

In this study, we focus on SST extremes in the coastal China Seas, which is one of the most sensitive regions to the global warming^35,36^. The cold extremes in the China Seas showed exceptional trend reversals when the last three decades are separated into the warming period (1982–1997) and hiatus period (1998–2013). These significant reverse patterns (i.e., from warming tendency to cooling tendency) mainly concentrated in the west of the Kuroshio Current, especially in the near-shore areas with shallow water depth, consistent with annual mean SST trends. It suggests that under the background of the global warming hiatus, cold extremes over the near-shore areas are much more sensitivity than in the open oceans. However, hot extremes trends exhibited non-significant warming across most part of the coastal China Seas. During global warming hiatus in this region there was an asymmetric pattern of greater cooling trends in cold SSTs as compared to the hot SSTs.

**Figure 6:**
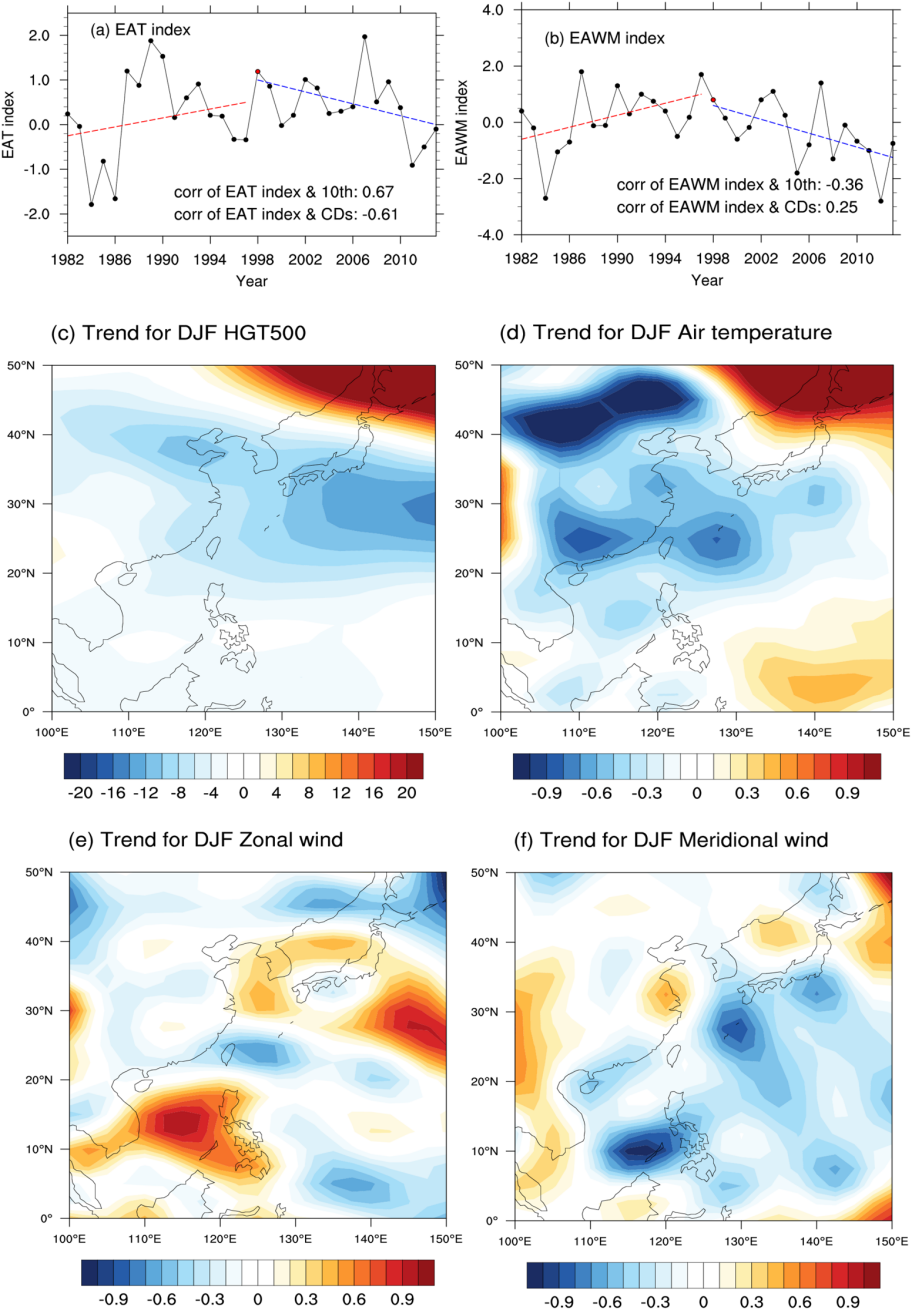
Time series of summer mean and 9-yr running mean (black lines) indices from 1982–2013 of the winter EAT index (**a**), EAWT index (**b**). Trend for 500-hPa geopotential height (**c**) (units: gpm decade^−1^), Trends for 1000-hPa air temperature (**d**) (units: 10*℃ decade^−1^), zonal wind (**e**) (units: 10* m s^−1^ decade^−1^), meridional wind (f) (units: 10* m s^−1^ decade^−1^) during 1982–2013 boreal winter (**c**); Data are at 2.5° resolution derived from NCEP/NCAR reanalysis. Maps on panels (**c**–**f**) were created by the authors using NCAR Command Language software Version 6.6.2 (see http://dx.doi.org/10.5065/D6WD3XH5).

Previous studies show that the global warming hiatus appears under the background of the two larger-scale circulations, Pacific Decadal Oscillation (PDO) and Artic Oscillation (AO) switch from positive phase to negative phase. PDO and AO in negative phase could release more cold air of the polar region into East Asia, intensify the East Asian winter monsoon (EAWM) and deepen of East Asian trough (EAT), especially for the interdecadal variability^12,37,38^. Actually, winter cold extremes in the China Seas are more easily affected by these local processes which may show more direct impacts^28,39^. The indices of EAT and EAWM in winter from 1982 to 2013 are presented in Fig. 6. The Pearson’s correlation coefficients between the EAWM index, the EAT index and cold extremes (including 10th SST and CDs) are calculated and shown in Fig. 6a,b. It is notable that the winter EAT index has been increasing during 1982 to 1997 and decreasing during 1998 to 2013. It means that the EAT was strengthening during 1998–2013. And the winter EAT index exerted a major influence with high significant correlations with 10th SSTs (*r* = 0.67, *p* < 0.01) and CDs (*r* = -0.61, *p* < 0.01), both exceeding 99% confidence level. As a moderate or weak impact, the EAWM index was only negatively correlated with 10th SSTs (*r* = − 0.36, *p* < 0.01), exceeding 95% confidence level. Then, the spatial trends of geopotential height at 500 hPa, air temperature, zonal wind and meridional wind at 1000 hPa are presented in Fig. 6. The geopotential height at 500 hPa has decreasing trends between 25°N and 40°N, suggesting that the EAT was deepening and westward extension. Meanwhile, the air temperature has also cooling trends over the whole China Seas. Although, the zonal wind showed heterogenous trends, the meridional wind decreasing significantly over most part of the China Seas, revealing that the northern wind is increasing. The atmosphere conditions support the hypothesis that the enhancing EAT shows more direct and significant impacts on SST by leading cold air southwards extension and strengthening cold waves. Cold waves can cool the upper ocean through the sensible heat flux and latent heat released from the ocean to the atmosphere which benefit for the outbreak of winter SST extremes.

Some local oceanic processes, for example upwelling currents, Kuroshio and ocean fronts also can impact the cold extremes^40–42^. The Kuroshio Heat Transport (KHT) through the PN section (‘PN’ stands for ‘Pollution Nagasaki’) from 1982 to 2013 is calculated using the method in Pei et al., 2017. Result shows that the KHT began rising in the early 1980s and continued until the late 1990s, and then began weakening since 1998 (figure omitted). The KHT is significantly correlated with winter cold extremes, 10th SST (r = 0.49, *p* < 0.01) and CDs (r = − 0.50, *p* < 0.01), both exceeding 99% confidence level. It is possible that there might be closely relationship between KHT and winter cold extremes in the China Seas. Coastal upwelling current is also an important driving mechanism for the SST change at northern SCS^43,44^. However, considering that atmospheric circulations are much notable factors in boreal winter and the limitation of paper space, only dynamical mechanisms of atmospheric modes were analyzed here. However, Further investigations on local oceanic processes are still needed.

## Method

Following with previous research^19,45^, our study focused on hot and cold SST extremes. To comprehensively investigate the characteristics of climate extremes, four extreme indices were calculated to represent the frequency and intensity properties of hot and cold SST extremes (shown in Table 1). We calculate the SST intensity exceeding/below fixed percentiles within each year. Here, for each pixel, annual hot extremes (SST_90p)_ are defined as the SST at the top 10% (90th percentile) of each year. In a similar way, annual cold extremes (SST_10p_) are defined as the SST at the bottom 10% (10th percentile) of each year. In case of temperature-based indices, the numbers of hot days (HDs) / cold days (CDs) are defined as the total number of days in each year with daily SST exceeding the 90th percentile/below 10th percentile of the baseline period of 1983–2012. The number of days exceeding or below such a threshold is the simplest way to reveal the change of frequency behavior and interpret the shift of the distribution in SST. As to this calculation method, HDs and CDs mainly occurred in the boreal summertime and wintertime, respectively.

The definition of EAT index adopted here is the one defined by Sun and Li (1997); that is, the EAT index is the normalized 500 hPa geopotential heights averaged over the area (25°–45°N, 110°–145°E). Note that in our study, when EAT index is lower than normal, EAT is deepened and strong, taking more cold air to Northwest Pacific Ocean, and vice versa. It is the same with EAWM index which are from the Chinese National Climate Center (CNCC). Monthly mean 500 hPa geopotential heights used to calculate EAT index in our study are from the National Centers for Environmental Prediction/National Center for Atmospheric Research (NCEP/NCAR) reanalysis (http://www.cdc.noaa.gov).

Our work was conducted on both a regional basis and a per-grid basis. Regional average is calculated by area-weighted averaging the grid data using latitude cosine as weights. Trends were calculated as the Sen’s estimator of the slope, which has been widely used in detecting monotonic trend in hydro-meteorological time series^46,47^. The nonparametric Mann–Kendall test was performed for the statistical significance test of trends. Note that we also obtained similar results by using the linear regression and Student’s t-test in this paper.
